# Veterinary–sanitary evaluation and biochemical quality of beef from cattle with chronic infectious diseases: Impact of chronic brucellosis on nutritional composition

**DOI:** 10.14202/vetworld.2026.1707-1723

**Published:** 2026-04-29

**Authors:** Leila Ansabayeva, Birzhan Nurgaliyev, Albina Darmenova, Yerbol Sengaliyev, Aigerim Kozhayeva, Altyn Zhubantayeva, Abzal Kenesovich Kereyev, Mira Nurgaliyeva

**Affiliations:** 1 Faculty of Agricultural Sciences, Akhmet Baitursynuly Kostanay Regional University, Kostanay, Kazakhstan; 2 Institute of Veterinary and Agrotechnology, West Kazakhstan Agrarian and Technical University named after Zhangir Khan, Uralsk, Kazakhstan; 3 Shakarim Laboratory, Shakarim University, Semey, Kazakhstan; 4 Institute of Industrial Technologies, West Kazakhstan University of Innovation and Technology, Uralsk, Kazakhstan

**Keywords:** beef quality, biochemical composition, brucellosis, cattle, fatty acid profile, meat safety, nutritional value, veterinary–sanitary assessment

## Abstract

**Background and Aim:**

Brucellosis is a chronic infectious disease of cattle that may influence not only animal health but also the nutritional and sanitary quality of meat. While veterinary–sanitary implications of infected carcasses are well documented, limited information is available on the biochemical composition of meat derived from chronically infected animals. This study aimed to evaluate the veterinary–sanitary status, organoleptic characteristics, and biochemical composition of beef obtained from cattle with chronic brucellosis compared with clinically healthy animals.

**Materials and Methods:**

An observational comparative cross-sectional study was conducted using post-slaughter samples collected within official veterinary surveillance programs. A total of 250 meat samples were subjected to veterinary–sanitary and organoleptic assessment, including animals diagnosed with brucellosis, leukemia, tuberculosis, and leptospirosis. Biochemical analysis was restricted to chronic brucellosis and matched controls (n = 100 per group). Standardized methods were used to determine proximate composition, mineral content, vitamin levels, fatty acid profile, and amino acid composition. Statistical analysis was performed using independent Student’s t-test, with significance set at p ≤ 0.05, and false discovery rate correction applied for multiple comparisons.

**Results:**

Veterinary–sanitary assessment revealed a higher proportion of carcass alterations and conditional suitability in infected animals compared with controls. Organoleptic evaluation indicated mild but consistent changes in color, texture, and overall quality of meat from infected cattle. Biochemical analysis demonstrated significant alterations in nutrient composition in the infected group, including reduced protein content and modifications in lipid fractions. Changes in fatty acid composition were observed, with variations in saturated and unsaturated fatty acids and altered polyunsaturated to saturated fatty acids and n-6 to n-3 ratios. Mineral and vitamin profiles also exhibited measurable differences between groups. Effect size analysis confirmed moderate to large differences for several key nutritional parameters, indicating biologically relevant impacts of chronic infection on meat quality.

**Conclusion:**

Chronic brucellosis is associated with measurable alterations in the biochemical composition and veterinary–sanitary quality of beef. Although meat from infected animals may remain conditionally suitable for consumption following regulatory assessment, its nutritional value can be compromised. These findings highlight the importance of integrating veterinary disease status into meat quality evaluation frameworks and support the need for continued surveillance and risk-based assessment in meat inspection systems.

## INTRODUCTION

In recent decades, issues related to the safety and quality of livestock products have become particularly relevant due to increasing consumer demands and international standards for the nutritional value, biological completeness, and sanitary reliability of meat raw materials [1]. Cattle meat is traditionally considered one of the most important sources of complete proteins, essential amino acids (EAA), vitamins, minerals, and fatty acids, which determine its nutritional and biological value [2]. However, the health of animals directly affects the chemical composition of muscle tissue, and chronic infections can significantly alter the quantitative and qualitative characteristics of meat, reducing its consumer and technological value [3, 4].

Chronic diseases such as leukemia, Brucella infection, tuberculosis (TB), and leptospirosis, which are common in cattle populations in many regions of the world, deserve special attention. These pathologies have a systemic effect on metabolism, causing inflammatory processes, immune dysfunction, and disturbances in the metabolism of proteins, fats, carbohydrates, and micronutrients. As a result, there is not only a decrease in the overall energy value of meat but also pronounced changes in amino acid composition and amino acid score (AAS), an imbalance in fatty acid content, and a deficiency of a number of vitamins and minerals. Such shifts may reduce the nutritional and biological value of meat and potentially influence dietary nutrient intake, rather than directly implying adverse health effects in consumers [5–8].

Current knowledge on brucellosis in cattle is predominantly focused on its epidemiology, zoonotic significance, and impact on reproductive performance and herd productivity. Veterinary–sanitary regulations provide clear guidance on carcass inspection and disposition; however, these frameworks are largely based on visible pathological lesions and public health risk, with comparatively limited emphasis on the biochemical and nutritional quality of meat derived from infected animals. Although several studies have evaluated meat quality traits under conditions such as metabolic disorders, stress, or parasitic infections, there remains a notable lack of comprehensive data examining how chronic brucellosis influences proximate composition, fatty acid profile, amino acid balance, and micronutrient content of beef. Furthermore, available reports often do not adequately control for confounding factors such as breed, age, production system, and body condition score (BCS), which can independently affect meat composition. Another important gap is the limited integration of veterinary–sanitary findings with biochemical evaluation, resulting in a fragmented understanding of how disease status translates into both regulatory classification and nutritional implications. In addition, most previous studies do not distinguish between acute and chronic infection stages, despite their potentially different physiological and metabolic impacts on muscle tissue. As a result, the extent to which chronic brucellosis alters meat quality beyond gross pathological assessment remains insufficiently characterized.

In this context, the present study aimed to provide an integrated evaluation of beef obtained from cattle with chronic brucellosis by combining veterinary–sanitary, organoleptic, and detailed biochemical analyses within a single comparative framework. Specifically, the study sought to (i) assess veterinary–sanitary outcomes and organoleptic characteristics of carcasses from infected and clinically healthy animals, (ii) quantify differences in proximate composition, mineral and vitamin content, fatty acid profile, and amino acid composition, and (iii) determine the statistical and biological significance of observed changes using a controlled and balanced study design. By restricting inferential biochemical analysis to chronic brucellosis and carefully matching animals for key production variables, the study aimed to minimize confounding and enhance the reliability of comparisons. Ultimately, this work intends to bridge the existing gap between veterinary inspection outcomes and nutritional quality assessment, thereby contributing to a more comprehensive understanding of how chronic infectious disease influences the safety, quality, and nutritional value of beef within modern meat production systems.

## MATERIALS AND METHODS

### Ethical approval

This study used muscle samples collected exclusively after routine commercial slaughter of cattle processed within official veterinary surveillance and meat inspection programs in Kazakhstan. No animals were experimentally infected, handled, restrained, or subjected to any additional invasive procedures for research purposes. Therefore, the study did not involve live-animal experimentation.

Because all specimens were obtained post-slaughter as part of routine commercial processing and official veterinary–sanitary control, formal institutional animal ethics committee approval was not required. Nevertheless, all procedures associated with ante-mortem inspection, slaughter, post-mortem examination, carcass handling, and sample collection were conducted in accordance with applicable national veterinary, food safety, and animal welfare regulations.

Animals included in the study were slaughtered only for routine commercial purposes and not specifically for this research. Sample collection was limited to tissues obtained after slaughter and did not influence animal management, transport, slaughter timing, or carcass disposition. The study therefore complied with the ethical principle of non-intervention in live animals.

Diagnostic classification of infected and control animals was based on routine veterinary surveillance data and standard diagnostic procedures already implemented within official herd health control programs. No additional diagnostic burden was imposed on animals for research purposes beyond those required under existing veterinary regulations.

All sampling and laboratory procedures were carried out with permission from the relevant slaughter and laboratory authorities, and data were recorded and analyzed in anonymized form. The study design, conduct, and reporting were aligned, where applicable, with internationally recognized reporting frameworks for observational veterinary research, including ARRIVE 2.0 principles for transparent animal research reporting and STROBE-Vet recommendations for observational studies in veterinary science.

### Study period and location

The study was conducted from September 2024 to September 2025 at the Faculty of Agricultural Sciences at Akhmet Baitursynov Kostanay Regional University. Meat from sick animals with brucellosis, TB, leukemia, and leptospirosis was collected from farms in Kostanay and neighboring regions of Kazakhstan for research. The quality of beef was evaluated at the Nutritest LLP laboratory in Kazakhstan.

### Sample collection

The object of the study was cattle meat. A total of 250 samples were analyzed for veterinary–sanitary examination and organoleptic assessment: 100 samples from animals diagnosed with brucellosis, 100 from animals with leukemia, and 25 samples each from animals with TB and leptospirosis. The smaller number of samples for TB and leptospirosis is explained by their lower prevalence during the study period. Veterinary and sanitary examination was conducted based on ante-mortem inspection and systematic post-mortem evaluation of carcasses and internal organs. Sanitary disposition was determined according to lesion distribution, systemic involvement, and general body condition.

Animals meeting inclusion criteria were selected consecutively at slaughter during the study period. No selection was performed based on carcass appearance, weight, or preliminary biochemical indicators, thereby minimizing selection bias.

Biochemical analyses were performed exclusively for chronic brucellosis and its matched control group. Other infections (TB, Bovine leukemia virus (BLV) infection, and leptospirosis) were included solely in the veterinary–sanitary and organoleptic assessment and were not pooled for biochemical comparison.

For the biochemical evaluation of meat in cases of brucellosis, a total of 200 samples were analyzed: 100 samples from clinically healthy animals (control group) and 100 samples from animals with chronic brucellosis infection. The diagnosis in animals was confirmed using established veterinary diagnostic methods in accordance with current guidelines and regulatory documents.

No pooled statistical comparison across different infectious diseases was performed. Each disease group was analyzed descriptively for veterinary–sanitary outcomes, while biochemical inference was restricted solely to chronic brucellosis versus matched controls.

Biochemical subgroup analysis for TB, BLV infection, and leptospirosis was not performed due to limited sample size (n = 25 per disease), which would not provide adequate statistical power for multivariable nutrient comparison. Therefore, inferential biochemical analysis was restricted to chronic brucellosis (n = 100), ensuring sufficient power and statistical reliability.

### Diagnosis and exclusion of co-infections

Brucellosis diagnosis: Brucellosis was diagnosed using a two-step serological approach. Initially, all animals were screened using the Rose Bengal Test (RBT) according to standard veterinary procedures. Samples showing visible agglutination were considered positive in the screening test. Positive samples were subsequently confirmed using a commercial indirect enzyme-linked immunosorbent assay (ELISA) for detection of antibodies against Brucella spp. Serum samples were considered positive when the sample-to-positive (S/P) ratio exceeded the manufacturer’s recommended cut-off value (e.g., ≥40% or optical density above the defined threshold, depending on the kit used). Only animals positive in both RBT and ELISA were classified as brucellosis-infected and included in the infected group.

Exclusion of bovine TB (bTB): BTB was excluded through routine intradermal tuberculin testing conducted within the official veterinary surveillance program. The single intradermal cervical test using purified protein derivative was performed, and animals showing a skin thickness increase of less than 4 mm without clinical signs were considered negative. Additionally, post-mortem inspection at slaughter did not reveal granulomatous lesions in lungs or lymph nodes suggestive of TB.

Exclusion of BLV infection: BLV infection was excluded using a commercial ELISA kit detecting antibodies against BLV gp51 antigen. Serum samples with S/P ratios below the manufacturer-defined threshold (e.g., <0.4) were classified as negative. Only BLV-negative animals were included in both study groups.

Exclusion of leptospirosis: Leptospirosis was excluded using the microscopic agglutination test following standard diagnostic protocols. Serum samples with titers below 1:100 were considered negative. Animals with titers equal to or above 1:100 were excluded from the study.

### Control group definition

Control animals were required to be negative for brucellosis (RBT and ELISA negative) and free from bTB, BLV infection, and leptospirosis according to the diagnostic procedures described above. Animals positive for more than one infectious agent were excluded from the study to avoid confounding effects of co-infections on biochemical parameters.

### Organoleptic and physicochemical assessment

All meat samples were subjected to organoleptic and physicochemical evaluation at the Institute of the Faculty of Agricultural Sciences at Akhmet Baitursynov Kostanay Regional University. Organoleptic and physicochemical properties were assessed in accordance with GOST 9959–2015 [9]. Assessment was qualitative and based on standardized visual and sensory examination. All assessments were performed by qualified laboratory personnel, with repeat evaluations conducted when necessary to ensure reliability.

### Selection of animals

Inclusion and exclusion criteria: The infected group included cattle serologically positive for brucellosis (RBT and ELISA positive) without severe cachexia (BCS ≥2.5) or systemic organ failure. Animals presenting advanced emaciation, co-infections, or evidence of TB or leptospirosis were excluded. This approach allowed the formation of a homogeneous group representing chronic active (compensated) brucellosis. Animals included in the infected group did not exhibit signs of terminal cachexia, severe anemia, organ failure, or advanced systemic deterioration. Post-mortem inspection confirmed absence of generalized purulent or necrotic lesions. Thus, the study population represents chronic compensated infection rather than terminal disease stages.

Chronic brucellosis was defined as seropositive status without acute febrile symptoms or terminal systemic deterioration. No animals exhibited signs of acute septicemia, severe organ failure, or advanced cachexia at the time of slaughter. Thus, the infected group represents chronic compensated infection rather than acute or terminal stages.

Sample size was determined based on the availability of animals meeting strict inclusion criteria during the study period. However, group sizes were consistent with similar comparative studies evaluating meat biochemical parameters.

Hematological parameters (where available from veterinary records), including hemoglobin concentration and leukocyte counts, did not indicate severe anemia or terminal inflammatory stages in included animals. Thus, the infected cohort represents chronic compensated infection rather than advanced cachectic disease.

Animal characteristics and production conditions: All animals included in the study were clinically examined prior to slaughter. Both control and infected groups consisted exclusively of female cattle of the Kazakh Whiteheaded breed in order to minimize genetic and sex-related variability in muscle composition.

The age of the animals ranged from 4 to 6 years, with no statistically significant difference between the control and infected groups (*p* > 0.05). Slaughter weight ranged from 420 to 480 kg and did not differ significantly between groups.

BCS was assessed using a standardized 5-point scale. Only animals with a BCS between 2.5 and 3.5 were included in the study to exclude severe cachexia or advanced emaciation that could independently influence meat composition.

All animals originated from extensive pasture-based production systems typical of the region. Feeding conditions were comparable between groups. The cattle were raised under natural grazing conditions without intensive feedlot finishing, and no differences in concentrate supplementation were recorded between groups.

Transport to the slaughterhouse did not exceed 2 h. Animals were not subjected to prolonged fasting prior to slaughter. Upon arrival, they were allowed a resting period of approximately 12 h with free access to water before slaughter.

Standard humane slaughter procedures were applied in accordance with national veterinary regulations. No signs of severe systemic disease, organ failure, or advanced emaciation were observed during ante-mortem or post-mortem inspection.

Age, breed, sex, production system, slaughter weight, and BCS were matched between groups to minimize confounding. No statistically significant differences in baseline animal characteristics were detected (p > 0.05).

Although formal a priori power calculation was not performed due to the observational design, post hoc power estimation based on the primary outcome (protein content difference of 5.5%, pooled standard deviation derived from standard error of the mean (SEM) values) indicated statistical power exceeding 0.80 at α = 0.05 for n = 100 per group.

The achieved statistical power (>0.80) indicates adequate sensitivity to detect moderate effect sizes (Cohen’s d ≥ 0.4) in primary biochemical outcomes.

### Sample collection and preparation

Samples were collected in accordance with the requirements [10]. Samples of the longest back muscle (m. longissimus dorsi), traditionally used in studies of the chemical composition and nutritional value of meat, were used for analysis. Samples weighing at least 200 g were cooled to a temperature of 0°C to +4°C, delivered to the laboratory, and prepared (cleaning of visible fat and connective tissue, grinding, and homogenization).

The time interval between slaughter and sample processing did not exceed 24 h. All samples were analyzed within 48 h after collection to minimize oxidative degradation and nutrient loss.

### Biochemical analysis

Determination of nutritional value. The mass fraction of protein was determined using the Kjeldahl method [11], fat using the Soxhlet method [12], moisture using the drying to constant weight method [13], and ash using the muffle furnace ashing method [14]. The energy value was calculated using Atwater coefficients and tables of the chemical composition of food products [15].

Determination of mineral composition. The content of sodium, potassium, calcium, magnesium, phosphorus, and iron was determined after mineralization of samples using atomic absorption spectrometry and photometry in accordance with methodological guidelines [16]. Limits of detection (LOD) and limits of quantification for mineral elements were determined according to standard calibration procedures and were within acceptable analytical ranges for meat matrix analysis.

Determination of vitamin composition. The concentration of vitamins A, E, B1, B2, niacin, and C was determined using spectrophotometric and chromatographic methods [16]. Method validation included assessment of linearity (R² > 0.995), repeatability, and recovery. LOD and quantification were established according to standard analytical procedures and were within acceptable ranges for meat matrix analysis.

Determination of fatty acid composition. Total lipids were extracted using a chloroform–methanol mixture (2:1, v/v) according to a modified Folch method prior to methyl ester preparation. The fatty acid profile was studied by gas chromatography of methyl esters of fatty acids [17]. The content of saturated fatty acids (SFA), monounsaturated fatty acids (MUFA), and polyunsaturated fatty acids (PUFA) was determined. Fatty acid methyl esters were analyzed using a gas chromatograph equipped with a flame ionization detector and a capillary column suitable for separation of long-chain fatty acids (e.g., SP-2560 or equivalent). Fatty acids were identified by comparison with certified reference standards based on retention times. In addition to individual fatty acids, total SFA, MUFA, PUFA, PUFA/SFA ratio, and n-6/n-3 ratio were calculated to provide comprehensive nutritional evaluation of lipid quality.

Determination of amino acid composition and AAS. The amino acid composition was determined by ion exchange chromatography after acid hydrolysis of the protein [18]. Protein hydrolysis was performed using 6N HCl at 110°C for 24 h under nitrogen atmosphere to prevent oxidative degradation. Tryptophan was determined separately following alkaline hydrolysis. The AAS was calculated as described previously [15], which involves comparing the content of EAA with the FAO/WHO reference profile. The AAS was calculated according to the FAO/WHO reference pattern for adults (FAO/WHO, 2007) as described previously [15]. The FAO/WHO (2007) adult amino acid requirement pattern was used as the reference scoring matrix, and calculations were performed per 100 g edible portion.

All biochemical analyses were performed in duplicate. Calibration of analytical instruments was carried out using certified reference materials prior to measurement. Inter-assay coefficient of variation did not exceed 5% for proximate composition and 7% for micronutrient analyses.

As this was an observational post-slaughter comparative study, causal relationships between infection status and biochemical alterations cannot be definitively established.

The study did not include direct microbiological or molecular detection of pathogens in muscle tissue. Therefore, conclusions are limited to nutritional composition and veterinary–sanitary classification rather than microbiological safety assessment.

Laboratory personnel performing biochemical analyses were blinded to infection status during sample processing and measurement to minimize analytical bias.

To facilitate practical interpretation, selected nutrient values were additionally expressed relative to international dietary reference intakes (RDA) per 100 g serving.

Internal laboratory quality control included analysis of certified reference meat materials with each analytical batch. Recovery rates ranged between 94% and 103%.

### Statistical analysis

Statistical processing of the data was performed using IBM SPSS Statistics v26.0 (IBM Corp., Armonk, NY, USA). All quantitative results are presented as mean ± SEM, where SEM reflects the precision of group estimates, while underlying variability and data distribution were additionally assessed during statistical diagnostics. The level of statistical significance was set at p ≤ 0.05. For comparative biochemical evaluation of meat in chronic brucellosis (n = 100 per group), independent two-sample Student’s t-tests (two-tailed) were applied to assess differences between clinically healthy and infected animals. Equal group sizes and matched baseline characteristics supported the robustness of parametric comparisons. Normality of distribution was assessed using the Shapiro–Wilk test, and homogeneity of variances was evaluated using Levene’s test. Outliers were screened using standardized residual analysis (z-scores > |3.0|); no biologically implausible extreme values requiring exclusion were detected, and all observations were within physiologically plausible ranges for bovine muscle tissue. Given the balanced group design and absence of substantial variance heterogeneity, parametric testing was considered appropriate. To account for multiple comparisons across proximate composition, mineral, vitamin, fatty acid, and amino acid profiles, false discovery rate (FDR) correction was applied using the Benjamini–Hochberg procedure. FDR adjustment was conducted within biologically related parameter groups (proximate composition, minerals, vitamins, fatty acids, and amino acids) to control type I error inflation while preserving statistical sensitivity. Adjusted q-values < 0.05 were considered statistically robust. In addition to p-values, effect sizes were calculated using Cohen’s d, with interpretation based on conventional thresholds (small: 0.2, moderate: 0.5, large: 0.8), to facilitate biological interpretation of the observed differences. Given the sample size (n = 100 per group), the study had sufficient statistical power (>0.80 at α = 0.05) to detect moderate and large effect sizes in primary biochemical outcomes. Categorical veterinary–sanitary outcomes (approval vs. condemnation) were summarized descriptively as proportions (%), as the objective of this component was epidemiological characterization rather than inferential modeling. Overall, the statistical framework combined hypothesis testing, multiple-comparison control, and effect size estimation to ensure statistical rigor and biological interpretability. Given the observational cross-sectional design, the identified differences should be interpreted as associations between infection status and meat composition parameters rather than direct causal effects.

All statistical assumptions were verified prior to hypothesis testing, and the analytical workflow was predefined to minimize type I error inflation and enhance reproducibility. Given the balanced design (n = 100 per group), the study had adequate power (>0.80) to detect moderate effect sizes, strengthening the robustness of the inferential conclusions.

## RESULTS

### Veterinary and sanitary assessment

**Brucellosis:** In cattle diagnosed with brucellosis, no significant macroscopic alterations were observed in skeletal musculature. Moderate enlargement of regional lymph nodes was detected in 32.0% of animals. Lesions were localized without multi-organ involvement. All carcasses (100%) were classified as conditionally fit for human consumption following mandatory heat treatment. No carcass condemnation was recorded in this group.

**bTB:** Among cattle positive for bTB, granulomatous lesions were identified primarily in the lungs and thoracic lymph nodes in 84.0% of animals. Caseous necrosis was detected in 48.0% of animals. Localized lesions were recorded in 72.0% of animals; these carcasses were conditionally approved after removal of affected tissues and heat treatment. Generalized lesions involving multiple organ systems were identified in 28.0% of animals, and these carcasses were condemned.

**BLV:** In cattle infected with BLV, subclinical presentation without gross pathological changes was observed in 80.0% of animals. Among the 80 subclinical animals, enlargement of lymph nodes was noted in 45 animals. Generalized neoplastic involvement was detected in 20.0% of animals. Carcasses without neoplastic lesions (80.0%) were classified as fit for human consumption without restrictions. Carcasses with generalized neoplastic involvement were condemned.

**Leptospirosis:** Among cattle diagnosed with leptospirosis, mild hepatic or renal alterations were observed in 40.0% of animals. No significant macroscopic muscle abnormalities were detected in localized forms. Carcasses without marked icterus or systemic pathology (88.0%) were conditionally approved after heat treatment. Pronounced icterus or generalized organ pathology was observed in 12.0% of animals, and these carcasses were condemned.

### Organoleptic evaluation

**Brucellosis:** Meat from brucellosis-positive cattle demonstrated acceptable appearance and characteristic aroma. Slight reduction in color intensity and taste expression was noted compared with control samples. Consistency showed a moderate decrease in firmness. Juiciness appeared slightly increased. No foreign or unacceptable odors were detected.

**bTB:** Samples from localized bTB cases exhibited reduced muscular fullness in some carcasses. The color on the cut surface appeared pale pink compared with controls. Taste intensity and broth richness were reduced. No putrid or abnormal odors were recorded.

**BLV:** Meat from BLV-infected cattle in subclinical stages did not demonstrate noticeable deviations in appearance, aroma, taste, or consistency compared with control samples.

**Leptospirosis:** In localized leptospirosis cases, organoleptic parameters remained within acceptable limits. In individual samples, moderate color variation and slight reduction in taste intensity were observed. No unacceptable sensory defects were detected in carcasses approved for processing. No ammonia odor, mold odor, rancidity, or other unacceptable sensory defects were identified in any approved carcasses.

**Comparative sanitary outcomes:** Carcass disposition differed depending on lesion dissemination. Generalized bTB (28.0%) and advanced neoplastic BLV infection (20.0%) represented the highest proportions of carcass condemnation within their respective disease groups. Brucellosis cases resulted exclusively in conditional approval after heat treatment. Mild leptospirosis cases were predominantly conditionally approved (88.0%). Subclinical BLV infection did not result in sanitary restrictions.

### Nutritional value of cattle meat in chronic infections (Brucellosis)

Chronic brucellosis significantly modified the proximate composition of beef (n = 100 per group), demonstrating a consistent reduction in nutrient density (Table 1). Protein content decreased by 5.50% in infected animals (18.56 ± 0.19 vs. 19.64 ± 0.39 g/100 g), with statistical significance maintained after FDR correction (p = 0.015; q = 0.019; d = 0.35, small–moderate). Fat content showed a more pronounced decline (−6.43%; p < 0.001; q < 0.001; d = 0.55, moderate), which was reflected in a corresponding 6.25% reduction in energy value (210 ± 2.5 vs. 224 ± 2.3 kcal/100 g; p < 0.001; q < 0.001; d = 0.58, moderate). The largest effect size was observed for ash content (−8.94%; p < 0.001; q < 0.001; d = 0.70, moderate–large), indicating a substantial decrease in the mineral fraction of muscle tissue. Moisture content increased slightly (+3.54%), although this change did not reach statistical significance (p = 0.091; q = 0.091; d = −0.24). Overall, chronic brucellosis was associated with statistically significant reductions in protein, lipid, mineral, and caloric values of beef compared with healthy controls. The slight increase in moisture content may be related to relative reduction of dry matter fraction and potential inflammatory tissue changes rather than true water accumulation.

**Table 1:** Nutritional value of cattle meat in cases of Brucellosis.

Parameters	Measurement	Healthy animals	Infected animals	The impact of chronic infections	p-value
Proteins	g/100 g	19.64 ± 0.39	18.56 ± 0.19	−5.50 %	0.015
Fats	g/100 g	16.17 ± 0.23	15.13 ± 0.14	−6.43 %	<0.001
Moisture	g/100 g	62.96 ± 1.04	65.19 ± 0.8	+3.54 %	0.091
Ash	g/100 g	1.23 ± 0.01	1.12 ± 0.02	−8.94 %	<0.001
Energy value	kcal/100 g	224 ± 2.3	210 ± 2.5	−6.25 %	<0.001

### Mineral composition of cattle meat in Brucellosis

Chronic brucellosis selectively affected the mineral profile of cattle meat (n = 100 per group), with significant reductions observed primarily in biologically critical micro- and macroelements (Table 2). No statistically significant differences were detected for sodium (−1.73%; p = 0.50; d = 0.10) or potassium (−2.02%; p = 0.42; d = 0.11), both demonstrating trivial effect sizes. Similarly, phosphorus showed only a minor, non-significant decline (−2.03%; p = 0.37; d = 0.13). These findings indicate relative stability of the principal intracellular electrolytes under chronic infection. In contrast, calcium content decreased significantly by 6.73% (9.7 ± 0.15 vs. 10.4 ± 0.08 mg; p < 0.001; q < 0.001), with a moderate effect size (d = 0.58). Magnesium was reduced by 6.09% (p = 0.027; q = 0.040; d = 0.32, small–moderate). The most pronounced alteration was observed for iron, which declined by 6.90% (2.7 ± 0.02 vs. 2.9 ± 0.03 mg; p < 0.001; q < 0.001), corresponding to a large effect size (d = 0.78). Importantly, all statistically significant findings remained robust after FDR correction (q < 0.05). The pattern of selective mineral depletion—particularly of iron and calcium— may indicate infection-associated metabolic redistribution of minerals rather than direct causal depletion associated with chronic brucellosis, potentially reflecting inflammation-driven alterations in mineral homeostasis and tissue redistribution. Overall, while major electrolytes remained stable, chronic brucellosis was associated with biologically meaningful reductions in key structural and hematologically relevant minerals, potentially diminishing the micronutrient value of beef.

**Table 2:** Mineral composition of cattle meat in Brucellosis.

Parameters	Measurement	Healthy animals	Infected animals	The impact of chronic infections	p-value
Sodium	mg	75.1 ± 0.9	73.8 ± 1.7	−1.73 %	0.50
Potassium	mg	356.3 ± 8.1	349.1 ± 3.4	−2.02 %	0.42
Calcium	mg	10.4 ± 0.08	9.7 ± 0.15	−6.73 %	<0.001
Magnesium	mg	23.0 ± 0.37	21.6 ± 0.5	−6.09 %	0.027
Phosphorus	mg	187.7 ± 3.6	183.9 ± 2.3	−2.03 %	0.37
Iron	mg	2.9 ± 0.03	2.7 ± 0.02	−6.90 %	<0.001

### Vitamin content in cattle meat in cases of chronic infections (Brucellosis)

Chronic brucellosis exerted a pronounced and systematic impact on the vitamin profile of cattle meat (n = 100 per group), with substantial reductions observed across both fat- and water-soluble vitamins (Table 3). The most marked decrease was detected for vitamin C (−30.77%; 0.9 ± 0.01 vs. 1.3 ± 0.03 mg), demonstrating an extremely large effect size (d = 1.79; p < 0.000001; q < 0.000001). Similarly, vitamin B1 declined by 27.27% (d = 1.18, very large), and vitamin B2 by 18.75% (d = 1.18, very large), both with highly significant p- and q-values (<0.000001). Fat-soluble antioxidants were also significantly reduced: vitamin A decreased by 16.67% (d = 1.26, very large), and vitamin E by 22.03% (d = 0.82, large), indicating marked impairment of antioxidant potential in infected animals. In contrast, niacin (PP) showed a modest, statistically non-significant decline (−7.38%; p = 0.16; q = 0.19; d = 0.20), suggesting relative metabolic stability of this vitamin under chronic infection. Importantly, all statistically significant differences remained robust after FDR correction (q < 0.05), confirming the reliability of the findings. The magnitude of the observed effect sizes, ranging from large to extremely large, indicates that chronic brucellosis profoundly compromises the vitamin composition of beef. This pattern likely reflects sustained inflammatory stress, oxidative imbalance, and altered metabolic turnover associated with chronic infection. Overall, the data demonstrate a biologically and nutritionally meaningful depletion of key antioxidant and metabolic vitamins in beef derived from brucellosis-affected cattle, substantially reducing its micronutrient value.

**Table 3:** Vitamin content in cattle meat in cases of Brucellosis.

Parameters	Measurement	Healthy animals	Infected animals	The impact of chronic infections	p-value
Vitamin A	µg	18 ± 0.2	15 ± 0.27	−16.67 %	<0.000001
Vitamin E	mg	0.59 ± 0.02	0.46 ± 0.01	−22.03 %	<0.000001
Vitamin B1	mg	0.11 ± 0.002	0.08 ± 0.003	−27.27 %	<0.000001
Vitamin B2	mg	0.16 ± 0.002	0.13 ± 0.003	−18.75 %	<0.000001
PP (Niacin)	mg	4.74 ± 0.20	4.39 ± 0.15	−7.38 %	0.16
Vitamin C	mg	1.3 ± 0.03	0.9 ± 0.01	−30.77 %	<0.000001

### Fatty acid content in cattle meat in cases of chronic infections (Brucellosis)

Chronic brucellosis markedly altered the fatty acid composition of beef (n = 100 per group), with a generalized reduction across SFA, MUFA, and PUFA (Table 4). Total fatty acids decreased by 7.46% (p = 0.001; q = 0.002; d = 0.46, moderate). SFA content showed a modest overall decline (−5.18%; p = 0.028; q = 0.040; d = 0.31, small–moderate). Within this fraction, myristic acid (C14:0) decreased substantially (−16.85%; d = 1.00, large), and margaric acid (C17:0) by 7.14% (d = 0.83, large), while palmitic (C16:0) and stearic (C18:0) acids showed only small, non-significant changes. MUFA exhibited a more pronounced reduction (−9.06%; p < 0.001; q < 0.001; d = 0.56, moderate). Palmitoleic acid (C16:1) declined by 17.76% (d = 1.24, very large), and oleic acid (C18:1), the predominant MUFA, decreased by 6.63% (d = 0.40, moderate), indicating a meaningful reduction in metabolically active lipid fractions. The most striking effects were observed in the PUFA fraction (−11.41%; p < 0.001; q < 0.001; d = 0.55, moderate). Linolenic acid (C18:3) decreased by 11.11% (d = 0.73, large), while arachidonic acid (C20:4) declined by 28.57% (d = 1.85, extremely large). Long-chain n-3 fatty acids showed dramatic depletion: eicosapentaenoic acid (C20:5) decreased by 35.29% (d = 2.14, extremely large), and docosahexaenoic acid (C22:6) by 46.67% (d = 3.95, massive). All statistically significant findings remained robust after FDR correction (q < 0.05). From a biological standpoint, the disproportionate reduction of long-chain PUFA, particularly n-3 fatty acids, suggests enhanced oxidative stress, altered lipid metabolism, and inflammatory remodeling of muscle tissue associated with chronic brucellosis. Nutritionally, these changes substantially diminish the functional lipid quality of beef, especially regarding cardioprotective and anti-inflammatory fatty acid fractions. Overall, chronic brucellosis is associated with a statistically robust and biologically profound deterioration of the fatty acid profile, with the most severe depletion observed in long-chain PUFA.

**Table 4:** Fatty acid content in cattle meat in cases of Brucellosis.

Parameters	Measurement	Healthy animals	Infected animals	The impact of chronic infections	p-value
Saturated fatty acid:	mg/100g	7047 ± 85.31	6682 ± 143.96	−5.18 %	0.028
C 14:0 Myristic acid	mg/100g	552 ± 12.28	459 ± 5.09	−16.85 %	<0.000001
C 16:0 Palmitic acid	mg/100g	4190 ± 72.65	4074 ± 75.81	−2.77 %	0.26
C 17:0 Margaric acid	mg/100g	266 ± 2.58	247 ± 1.95	−7.14 %	<0.000001
C 18:0 Stearic acid	mg/100g	2039 ± 41.83	1952 ± 24.60	−4.27 %	0.07
Monounsaturated fatty acid:	mg/100g	8428 ± 151.69	7664 ± 120.57	−9.06 %	<0.001
C 16:1 Palmitoleic acid	mg/100g	991 ± 12.76	815 ± 15.49	−17.76 %	<0.000001
C 18:1 Oleic acid	mg/100g	7174 ± 148.30	6698 ± 81.45	−6.63 %	0.004
C 20:1 Gadoleic acid	mg/100g	163 ± 1.39	151 ± 3.04	−7.36 %	0.001
Polyunsaturated fatty acid:	mg/100g	631 ± 16.28	559 ± 7.09	−11.41 %	<0.001
C 18:2 Linoleic acid	mg/100g	409 ± 4.81	376 ± 6.66	−8.07 %	<0.001
C 18:3 Linolenic acid	mg/100g	162 ± 2.99	144 ± 1.88	−11.11 %	<0.000001
C 20:4 Arachidonic acid	mg/100g	28 ± 0.39	20 ± 0.47	−28.57 %	<0.000001
C 20:5 Eicosapentaenoic acid	mg/100g	17 ± 0.35	11 ± 0.17	−35.29 %	<0.000001
C 22:6 Docosahexaenoic acid	mg/100g	15 ± 0.23	8 ± 0.06	−46.67 %	<0.000001
Total fatty acids	mg/100g	16106 ± 176.43	14905 ± 322.62	−7.46 %	0.001

### Amino acid content in cattle meat during chronic infections (Brucellosis)

Chronic brucellosis significantly altered the amino acid composition of beef (n = 100 per group), with a general decline in total amino acid content (−5.40%; p = 0.03; q = 0.045; d = 0.30, small–moderate) (Table 5). Total EAA decreased by 7.24% (p < 0.001; q < 0.001; d = 0.56, moderate), indicating reduced biological protein value. Among EAAs, methionine showed the most dramatic depletion (−31.06%; d = 2.34, extremely large), followed by threonine (−15.33%; d = 1.03, large), valine (−10.70%; d = 0.62, moderate), and leucine (−8.52%; d = 0.44, moderate). Tryptophan exhibited a modest but significant decline (−7.21%; d = 0.39). In contrast, isoleucine increased by 8.59% (d = −0.75, moderate–large), suggesting a selective metabolic redistribution. Lysine and phenylalanine remained statistically unchanged. Total non-EAA (NEAA) decreased by 4.23% (p = 0.011; q = 0.015; d = 0.36, small–moderate). Marked reductions were observed for histidine (−23.87%; d = 1.84, extremely large), cysteine (−10.62%; d = 1.29, very large), glutamic acid (−9.83%; d = 0.55, moderate), asparagine (−8.23%; d = 0.50, moderate), tyrosine (−10.36%; d = 0.51, moderate), and serine (−5.22%; d = 0.44, moderate). Conversely, proline (+21.99%; d = −1.29, very large) and hydroxyproline (+14.71%; d = −1.37, very large) increased substantially, alongside a moderate rise in arginine (+7.27%; d = −0.40). These changes suggest enhanced connective tissue deposition and altered collagen metabolism in chronically infected animals. All statistically significant differences remained robust after FDR correction (q < 0.05). Collectively, the data demonstrate that chronic brucellosis leads to a measurable deterioration of protein quality in beef, characterized by depletion of key EAA, particularly sulfur-containing and regulatory amino acids, alongside structural remodeling reflected by increased collagen-associated fractions.

**Table 5:** Amino acid content in cattle meat in cases of Brucellosis.

Parameters	Measurement	Healthy animals	Infected animals	The impact of chronic infections	p-value
Total essential amino acids	mg/100g	7537 ± 106.57	6991 ± 87.76	−7.24 %	<0.001
Valine	mg/100g	1093 ± 21.81	976 ± 15.18	−10.70 %	<0.001
Isoleucine	mg/100g	826 ± 9.17	897 ± 9.63	+8.59 %	<0.000001
Leucine	mg/100g	1561 ± 26.75	1428 ± 32.97	−8.52 %	0.002
Lysine	mg/100g	1678 ± 39.22	1615 ± 14.91	−3.75 %	0.15
Methionine	mg/100g	470 ± 4.40	324 ± 7.55	−31.06 %	<0.000001
Threonine	mg/100g	848 ± 13.98	718 ± 11.13	−15.33 %	<0.000001
Tryptophan	mg/100g	222 ± 4.99	206 ± 2.12	−7.21 %	0.005
Phenylalanine	mg/100g	839 ± 10.65	827 ± 16.04	−1.43 %	0.52
Total non-essential amino acids	mg/100g	11923 ± 103.61	11419 ± 169.98	−4.23 %	0.011
Alanine	mg/100g	1147 ± 19.29	1115 ± 15.33	−2.79 %	0.20
Arginine	mg/100g	1101 ± 12.42	1181 ± 23.60	+7.27 %	0.004
Asparagine	mg/100g	1870 ± 37.80	1716 ± 21.33	−8.23 %	<0.001
Histidine	mg/100g	750 ± 8.75	571 ± 10.69	−23.87 %	<0.000001
Glycine	mg/100g	989 ± 21.48	1029 ± 8.46	+4.04 %	0.09
Glutamic acid	mg/100g	3245 ± 43.35	2926 ± 69.37	−9.83 %	<0.001
Oxiproline	mg/100g	306 ± 2.81	351 ± 3.72	+14.71 %	<0.000001
Proline	mg/100g	723 ± 10.13	882 ± 14.10	+21.99 %	<0.000001
Serine	mg/100g	824 ± 11.07	781 ± 8.52	−5.22 %	0.002
Tyrosine	mg/100g	695 ± 14.58	623 ± 13.41	−10.36 %	<0.001
Cysteine	mg/100g	273 ± 2.64	244 ± 1.66	−10.62 %	<0.000001
Total amino acids	mg/100g	19460 ± 368.94	18410 ± 325.17	−5.40 %	0.03

### AAS of cattle meat in chronic infections (Brucellosis)

Chronic brucellosis significantly modified the AAS profile of beef proteins (n = 100 per group), indicating alterations in biological protein quality relative to reference requirements (Table 6). The most pronounced decline was observed for methionine + cystine (−19.44%; 87% vs. 108%; p < 0.000001; q < 0.000001), with an extremely large effect size (d = 2.39). This shift identifies sulfur-containing amino acids as the limiting fraction in infected animals, representing a critical reduction in protein biological value. Threonine (−10.19%; d = 0.55, medium) and valine (−5.41%; d = 0.76, medium–large) also showed significant decreases (q < 0.001), whereas leucine exhibited a small, non-significant decline. Tryptophan and lysine remained statistically stable, and phenylalanine + tyrosine showed no change (d = 0.00). In contrast, isoleucine increased significantly (+11.02%; d = −0.77, medium–large; q < 0.000001), suggesting selective metabolic redistribution rather than uniform protein depletion. All statistically significant findings remained robust after FDR correction. Overall, chronic brucellosis leads to a deterioration of protein quality in beef, primarily due to a substantial reduction in sulfur-containing amino acid adequacy, thereby lowering the limiting AAS and potentially diminishing the nutritional and functional value of the protein fraction.

**Table 6:** Amino acid score of cattle meat in Brucellosis.

Parameters	Measurement	Healthy animals	Infected animals	The impact of chronic infections	p-value
Isoleucine	%	118 ± 2.01	131 ± 1.27	+11.02 %	<0.000001
Leucine	%	114 ± 1.39	110 ± 2.28	−3.51 %	0.14
Lysine	%	155 ± 3.13	158 ± 2.05	+1.94 %	0.44
Methionine + Cystine	%	108 ± 1.05	87 ± 0.65	−19.44 %	<0.000001
Phenylalanine + Tyrosine	%	130 ± 1.51	130 ± 2.20	–	1.00
Threonine	%	108 ± 2.64	97 ± 1.13	−10.19 %	<0.001
Tryptophan	%	113 ± 1.50	111 ± 2.32	−1.77 %	0.50
Valine	%	111 ± 0.81	105 ± 0.77	−5.41 %	<0.000001
Limiting amino acid score	%	–	–	–	–

## DISCUSSION

### Veterinary and sanitary assessment

Unlike previous studies that primarily focused on sanitary inspection or basic proximate composition, the present study provides an integrated veterinary–sanitary, organoleptic, and multi-level biochemical characterization of beef under naturally occurring chronic brucellosis in a controlled matched production system.

In our study, veterinary–sanitary outcomes varied depending on the extent and dissemination of pathological lesions observed at slaughter. Veterinary–sanitary outcomes differed according to the degree of systemic involvement. In brucellosis, the absence of significant muscular lesions and the predominance of localized lymph node enlargement resulted in 100% conditional approval after heat treatment, with no carcass condemnation.

In contrast, bTB showed a higher frequency of granulomatous and caseous lesions, and 28% of animals had generalized pathology requiring condemnation, confirming its greater sanitary significance. For BLV infection, most cases were subclinical and carcasses were approved without restrictions; however, generalized neoplastic involvement (20%) led to condemnation.

In leptospirosis, lesions were mainly mild and organ-limited, with 88% of carcasses conditionally approved and only severe icteric or generalized cases rejected. Overall, carcass disposition was primarily determined by lesion dissemination, with TB presenting the highest risk of full condemnation.

Other studies have demonstrated comparable sanitary–pathological patterns across chronic infectious diseases of cattle.

**Brucellosis:** Brucellosis is a major zoonosis; meat from infected animals can carry Brucella and pose risk to workers and consumers, especially if eaten raw or undercooked [19, 20]. Infected animals are often not distinguishable by gross carcass lesions. In a large Brazilian slaughterhouse study, 9.8% of cattle were seropositive for brucellosis, yet no macroscopic post-mortem changes suggestive of brucellosis were detected; main findings were non-specific (contamination, blood aspiration, emphysema) [21]. Classical lesions that, when present, may raise suspicion include bursitis, arthritis, hygromas, orchitis/epididymitis, metritis, purulent genital discharges and udder lesions, but these are rare in slaughter cattle [21]. In camels, about 9.4% were seropositive; nearly half of carcasses had lesions leading to partial or total condemnation (pericarditis, hepatomegaly with nodular lesions, enteritis, visceral hemorrhages), but these lesions can reflect brucellosis or other zoonoses [22]. Cervical bursitis in cattle is a recognized “brucellosis-suggestive” lesion; in such carcasses, serology (Rose Bengal, SAT/2-ME) has high sensitivity and good specificity and can guide sanitary decisions [23]. Overall, reliable detection at the abattoir depends on laboratory testing (serology, culture/polymerase chain reaction [PCR]), not on organoleptic or gross changes alone [21, 23–25].

**TB:** Across abattoir studies, post-mortem inspection for bTB relies primarily on gross (macroscopic) and organoleptic assessment of carcasses and offal, followed by laboratory confirmation where available. Routine meat inspection commonly includes visual inspection, palpation and selective incisions of lungs, liver, gastrointestinal tract and key lymph nodes (especially tracheobronchial, mediastinal, prescapular/precrural) to detect TB-like lesions [26–29]. When miliary or generalized bTB involving multiple lymph nodes or organs is present, the whole carcass is condemned; localized lesions lead to partial or whole-organ condemnation [26, 29]. Detailed (complete) meat inspection, with systematic incision of a wide set of lymph nodes (parotid, retropharyngeal, mesenteric, iliac, supramammary, etc.) and organs at 2-cm intervals under bright light, detects up to 3- to 8-fold more bTB-positive carcasses than routine inspection and is therefore considered the sanitarily safer standard [26, 29, 30]. However, several studies show that even detailed gross inspection can still miss carcasses with microscopic lesions or culture/PCR-confirmed infection [31–34].

**BLV:** Research links BLV infection with distinct changes in meat quality, especially at the hematological (clinically sick) stage. These changes are central to veterinary–sanitary examination and organoleptic assessment [35]. Veterinary–sanitary examination includes: Pre-slaughter diagnostics: serology (RID, ELISA) and hematology to detect BLV-infected and leukemic animals; ELISA is more sensitive than RID when applied to carcass washings and by-products [36]. Post-slaughter assessment: organoleptic checks (appearance, color, drying crust, consistency, odor, broth properties) physicochemical tests (pH, peroxidase reaction, moisture, fat, ash, caloric value) [37, 6, 38]; bacterioscopic/ microbiological examination of muscles and organs [3, 6]. Current data lead to the veterinary–sanitary conclusion that meat from cattle in the hematological stage of leukemia does not correspond to “fresh” meat and, due to degraded nutritional value, microbial load, and presence of heat-resistant toxic metabolites, should be excluded from the human food chain [6, 39].

**Leptospirosis:** Leptospirosis is a major zoonosis; pathogenic *Leptospira* can be present in blood, kidneys, liver, urine and genital tract of cattle, goats, sheep and pigs at slaughter, often in the absence of clear clinical signs [40–44]. This creates a direct food safety and occupational risk for slaughterhouse workers and consumers of undercooked meat or offal [42, 43, 45, 46].

In cattle and small ruminants, leptospirosis can be acute (septicemic) or chronic. Acute disease produces multisystemic lesions affecting key edible organs: kidneys (nephritis), liver (hepatomegaly, congestion), spleen (splenomegaly), and hemorrhages in lungs and heart [44, 47, 48]. Pathological findings in calves include kidney alterations, icterus, congested or enlarged liver, splenomegaly, and petechiae on heart and lungs; adult carcasses may show placentitis, necrosis, vasculitis and visible leptospires histologically [44].

These lesions may be accompanied by jaundice and poor body condition and can alter color and appearance of meat and organs, but systematic descriptions of smell, texture or taste are not provided.

It is important to emphasize that the present study did not include microbiological or molecular detection of pathogens in muscle tissue. Therefore, the results should not be interpreted as direct evidence of microbiological safety, but rather as an assessment of sanitary classification and nutritional quality within regulated veterinary inspection frameworks. Accordingly, the study focuses on nutritional and sanitary classification aspects and does not aim to evaluate zoonotic transmission risk through meat consumption.

### Organoleptic evaluation

In our study, organoleptic changes were generally mild and depended on the type and severity of infection.

In brucellosis, meat retained acceptable sensory characteristics, with only slight reductions in color intensity, firmness, and taste expression, while remaining free from abnormal odors.

In localized bTB, moderate deterioration in muscular fullness and taste intensity was observed, although no putrid or unacceptable odors were detected.

Meat from subclinical BLV-infected cattle did not differ noticeably from control samples, indicating minimal impact at early stages.

Similarly, in localized leptospirosis, sensory parameters largely remained within acceptable limits, with only minor variations in color and taste.

Importantly, no approved carcasses exhibited signs of spoilage such as ammonia odor, mold, or rancidity, confirming that sensory deviations were moderate and primarily associated with disease-related metabolic alterations rather than microbial deterioration.

Other studies have demonstrated comparable organoleptic patterns in cattle affected by chronic infectious diseases, indicating that sensory deviations are generally mild in localized or subclinical stages and become pronounced mainly with systemic or advanced pathology.

**Brucellosis:** A focused comparison of meat from cows seropositive for brucellosis vs healthy controls reported: Lower fat content in brucellosis-positive cows (11.0% vs 14.1%; 1.28-fold decrease) due to poorer body condition [5].

Lower protein content (15.9% vs 19.2%; 1.2-fold decrease), and reduced tryptophan and oxyproline, indicating somewhat reduced nutritional and protein quality indices [5].

Moisture and ash contents were similar; overall biochemical and physicochemical differences were described as moderate, not drastic [5].

Authors note that, despite systemic metabolic disturbance in brucellosis, the deviations in meat quality indicators were not marked compared with healthy cows [5].

Organoleptically, the literature here does not describe specific changes in color, odor, or taste unique to brucellosis; the main effect is reduced fatness and therefore potentially leaner, slightly less marbled meat [5, 21].

**TB:** Meat and organs with tuberculous lesions typically show: Localized or multiple tubercles/granulomas, often in lungs and thoracic lymph nodes (mediastinal, tracheobronchial), but also head, mesenteric and other lymph nodes, liver, spleen and intestines [27, 30, 31, 34, 49].

Lesions described as nodules or abscesses with a necrotic, caseous (“cheesy”) or caseopurulent center, sometimes with calcification and surrounded by a fibrous capsule [27–30].

Variable size, color and consistency depending on stage: from small, firm gray-white nodules to large fibro-caseous or calcified masses that may deform or partly replace the organ [27].

In generalized disease, multiple affected organs and lymph nodes, often with miliary lesions, render the carcass unfit for human consumption [26, 29, 34].

Importantly, a large proportion of infected animals show no visible lesions at slaughter (asymptomatic or early infection), so carcasses may appear organoleptically normal yet be infected [31, 33, 50]. Across settings, routine visual inspection alone shows low sensitivity (roughly 20–30% vs. more sensitive tests) but high specificity [26, 28, 29]. Molecular and culture methods (PCR, GeneXpert, histopathology, IFN-γ tests) greatly improve detection and are recommended as ancillary tools for sanitary control, particularly where bTB is endemic [27, 28, 32, 33].

**BLV:** Meat from cattle killed in the hematological stage shows marked deviations from normal sensory characteristics: Consistency and elasticity: reduced elasticity; finger impressions level slowly, indicating loss of turgor [35]. Color: abnormal color of muscle tissue compared with healthy animals [35]. Surface condition: in 87% of carcasses only a slightly developed drying crust is formed, suggesting high surface moisture and poorer keeping quality [35]. Bouillon quality: broth becomes turbid with sour, unpleasant odor, not typical of fresh meat [35].

A more recent biochemical study confirms parallel quality loss: increased moisture (≈+2.7%), reduced protein and fat, and lowered caloric value in meat from BLV-infected and especially clinically sick animals, all detrimental to flavor, processing, and shelf life [6].

**Leptospirosis:** The literature focuses on epidemiology and diagnostics rather than detailed organoleptic descriptions. Acute leptospirosis in calves can cause icterus, congested liver, hepatomegaly, splenomegaly, petechiae in lungs and heart, and pathological kidney changes [44]. Such lesions may be associated with: Yellow discoloration of carcass and organs (jaundice). Congested, enlarged liver and spleen. Petechial hemorrhages on serosal surfaces and in lungs/heart.

However, many infected slaughter animals are chronic carriers with no specific gross or organoleptic abnormalities, so absence of visible changes does not guarantee safety [40–42, 44].

### Effects of brucellosis on cattle meat biochemistry

The present findings should be interpreted primarily in the context of chronic compensated brucellosis under field conditions, rather than acute or terminal disease stages.

Chronic brucellosis was associated with a consistent decline in the nutritional quality of beef. Moderate reductions in protein, fat, ash, and energy value indicate decreased nutrient density, accompanied by selective depletion of biologically important minerals, particularly iron and calcium.

More pronounced alterations were observed in the vitamin and lipid fractions, with substantial losses of antioxidant vitamins (A, E, C, B1, and B2) and marked depletion of long-chain PUFA, especially n-3 fractions (EPA and DHA). From a nutritional perspective, the disproportionate reduction of long-chain PUFA, particularly n-3 fatty acids, indicates a substantial decline in the functional lipid quality of beef derived from chronically infected animals.

Protein quality was further compromised by reductions in EAA, most notably methionine and cysteine, resulting in a lower AAS and identification of sulfur-containing amino acids as the limiting fraction. Concurrent increases in proline and hydroxyproline indicate connective tissue remodeling and altered muscle structure.

Overall, chronic brucellosis leads not only to quantitative nutrient loss but to structural biochemical remodeling of muscle tissue, significantly diminishing the biological and functional value of beef.

The only meat-focused study compares meat from seropositive vs. healthy cows across basic biochemical traits: dry matter, protein, fat, volatile fatty acids, amino-ammonia nitrogen, tryptophan, hydroxyproline, and a protein quality index [5].

Brucellosis-positive cows had lower protein (19.2 → 15.9%), fat (14.1 → 11.0%), dry matter, VFAs, tryptophan, and PQI than healthy animals [5]. Moisture and ash were similar (Agoltsov et al., 2021). Authors attribute this mainly to poor body condition and reduced feed intake in sick cows rather than a specific tissue defect [5].

No available studies provide detailed mineral profiles, vitamin composition, fatty acid composition, individual amino acid profiles, or AAS of cattle meat specifically affected by brucellosis. Existing biochemical work on brucellosis focuses on serum proteins and minerals (e.g., Na, Ca, Mg, K) rather than muscle tissue [51–54].

Current research shows modest reductions in protein, fat, and a general protein quality index in meat from brucellosis-positive cows, largely linked to poorer nutritional status. Detailed data on mineral, vitamin, fatty acid, and amino acid composition or AAS of such meat have not been characterized in the literature found here.

### Systemic biochemical changes in brucellosis

Multiple studies show consistent biochemical disturbances in infected cattle: Liver and muscle enzymes: aspartate aminotransferase and alanine aminotransferase are commonly increased, reflecting hepatic/muscle damage [52–57]. Creatine kinase can also rise [55].

**Renal/other metabolites:** Creatinine often ↑ or unchanged; urea usually unchanged or mildly affected [52–56].

Lipids and proteins: Cholesterol and triglycerides frequently ↑; total protein may ↑ with altered albumin/globulin balance (albumin ↓, globulin ↑ or vice versa depending on stage/comorbidity) [51, 52, 54, 55, 57, 58].

**Minerals:** Serum Fe tends to ↓ [53]; Na, Ca, Mg may ↑, K ↓ in some herds [51].

**Oxidative stress:** Increased malondialdehyde (MDA) and nitric oxide, decreased antioxidant defenses such as glutathione and key enzymes indicate oxidative stress [53, 59].

These systemic changes help explain modest reductions in meat protein and fat and support using biochemical profiling to gauge disease impact and organ involvement [5, 52–56, 58].

To the best of our knowledge, this is one of the first studies in Central Asia and internationally to provide a comprehensive evaluation of mineral, vitamin, fatty acid, amino acid composition, and AAS of beef derived from cattle with chronic brucellosis under naturally occurring field conditions.

Brucellosis in cattle leads to mild–moderate deterioration of meat biochemical quality (notably lower protein and fat) and clear systemic biochemical and oxidative stress changes in blood, mainly indicating liver, muscle, and metabolic dysfunction.

## CONCLUSION

The present study demonstrated that chronic brucellosis is associated with measurable and consistent alterations in both veterinary–sanitary classification and the biochemical composition of beef. All carcasses from brucellosis-affected animals were conditionally approved following heat treatment, with no cases of condemnation, indicating limited gross pathological involvement compared with other infections. Organoleptic evaluation showed generally acceptable sensory quality, although slight reductions in color intensity, taste expression, and firmness were observed. Importantly, detailed biochemical analysis revealed significant reductions in protein (−5.50%), fat (−6.43%), ash (−8.94%), and energy value (−6.25%), along with substantial depletion of key micronutrients, including calcium, magnesium, and iron. The most pronounced changes were observed in vitamin composition, particularly vitamin C, vitamin B1, and vitamin E, as well as in fatty acid profiles, where long-chain PUFA were markedly reduced. Amino acid analysis further confirmed deterioration in protein quality, with significant depletion of EAA, especially methionine, and a reduction in AAS due to limiting sulfur-containing amino acids.

From a practical perspective, these findings indicate that although meat from chronically infected animals may remain conditionally suitable for consumption under veterinary regulations, its nutritional value is compromised. The reduction in essential nutrients, antioxidant vitamins, and biologically important fatty acids suggests potential implications for human nutrition, particularly in populations relying heavily on meat as a primary nutrient source. Therefore, integration of disease status into meat quality evaluation systems and regulatory frameworks may be warranted to ensure both safety and nutritional adequacy.

A key strength of this study is the comprehensive and integrated approach combining veterinary–sanitary assessment, organoleptic evaluation, and detailed biochemical profiling within a controlled comparative design. The use of matched groups, standardized analytical methods, and statistical correction for multiple comparisons enhances the reliability and robustness of the findings. Additionally, the focus on chronic brucellosis provides valuable insight into the long-term metabolic impact of infection on meat quality, which has been insufficiently explored in previous studies.

However, several limitations should be considered. The observational cross-sectional design does not allow establishment of causal relationships between infection and biochemical alterations. Biochemical analyses were restricted to chronic brucellosis, and other infectious diseases were not evaluated at the same level of detail due to limited sample size. Furthermore, the study did not include direct microbiological or molecular assessment of muscle tissue, and therefore conclusions are limited to nutritional and sanitary aspects rather than microbiological safety.

Future research should focus on longitudinal and mechanistic studies to elucidate the pathways underlying nutrient depletion in infected animals, including the role of inflammation, oxidative stress, and metabolic redistribution. Expanded studies involving multiple breeds, production systems, and geographic regions would improve generalizability. In addition, integrating microbiological safety assessment with nutritional evaluation would provide a more comprehensive framework for meat quality assessment in diseased animals.

In conclusion, chronic brucellosis significantly affects the biochemical and nutritional quality of beef despite acceptable veterinary–sanitary classification. These findings highlight the need for a more integrated approach to meat evaluation that considers not only safety and regulatory compliance but also nutritional integrity, thereby supporting improved public health and food quality standards.

## DATA AVAILABILITY

The data generated during the study are included in the manuscript.

## AUTHORS’ CONTRIBUTIONS

LA, BN, AD, and YS: Conceived and designed the study. YS, AZ, AKK, and MN: Conducted sample collection and laboratory analyses. LA, AKK, BN, and AK: Performed data analysis and interpretation. AD, AZ, MN, and AK: Drafted the manuscript. All authors have critically revised the manuscript and approved the final version of the manuscript.
